# Is Working Memory Training Effective? A Study in a School Setting

**DOI:** 10.1371/journal.pone.0104796

**Published:** 2014-08-27

**Authors:** Catrin Rode, Robby Robson, Andy Purviance, David C. Geary, Ulrich Mayr

**Affiliations:** 1 Department of Psychology, University of Oregon, Eugene, Oregon, United States of America; 2 Eduworks, Corvallis, Oregon, United States of America; 3 Psychological Sciences, University of Missouri, Columbia, Missouri, United States of America; University Medical Center Groningen UMCG, Netherlands

## Abstract

We tested the effectiveness of an intensive, on average 17-session, adaptive and computerized working-memory training program for improving performance on untrained, paper and pencil working memory tasks, standardized school achievement tasks, and teacher ratings of classroom behavior. Third-grade children received either a computerized working memory training for about 30 minutes per session (*n* = 156) or participated in regular classroom activities (*n* = 126). Results indicated strong gains in the training task. Further, pretest and posttest transfer measures of working memory and school achievement, as well as teacher ratings, showed substantial correlations with training task performance, suggesting that the training task captured abilities that were relevant for the transfer tasks. However, effect sizes of training-specific transfer gains were very small and not consistent across tasks. These results raise questions about the benefits of intensive working-memory training programs within a regular school context.

## Introduction

The attentional and inhibitory control components of working memory (WM), also termed the central executive, are limited mental resources that support the maintenance and integration of information in the service of problem solving and learning [1], [2]. Working memory capacity is typically assessed with tasks that combine a storage and processing demands and are often referred to as “complex span” tasks [3]. A large and consistent body of research shows that individual differences in these tasks predict individual differences in a wide array of more complex cognitive tasks such as typical fluid intelligence and problem solving measures [4], [5]. Moreover, working memory capacity has proven to be a strong predictor of mathematics and reading achievement and across-grade gains in achievement [6]–[9]. Given this powerful relationship it is a plausible hypothesis that if one were to find a way to increase students' working memory capacity this should have wide-ranging benefits for intellectual and academic functioning.

Until recently, however working memory capacity has been considered to be a relatively stable individual differences trait [10]. As a result, remediation of academic deficits for children with below average working memory capacity focused on explicit, content-specific strategies that enable optimal use of limited working memory resources [11]–[13]. However, more recently there has been some evidence that, through an intensive adaptive training regimen, working memory capacity itself can be increased [14]. If this is correct, improved working memory capacity should translate into wide-ranging benefits, especially for those students with poor WM functions. There is in fact some evidence of gains on measures of fluid intelligence and academic achievement following working memory training [15]–[18]. At this point, it is however not clear to what degree such a core working memory training program can be embedded within a regular school context and to what degree it produces benefits on academically relevant abilities that exceed those of regular class participation. Therefore, the main goal of the current project was to adapt an existing training program [19] to and test ifs effectiveness within a relatively large sample of 3^rd^ grade students within a classroom context.

### Relationships Among Training and Transfer Tasks

In evaluating the potential of working memory training for boosting childrens' academic performance and learning it is important to consider a number of unresolved questions [20]. One particularly important issue is that most existing training studies have only documented transfer on individual tasks, without examining the relationship among transfer tasks. In the current study, we attempted to capture transfer of training effects across a diverse set of six measures, which varied on several dimensions such as computerized versus paper-and-pencil testing, near versus far transfer (working memory versus standard achievement tests), immediate versus delayed testing, and objective assessments versus teacher rating. The diversity of these measures is a reflection of both the constraints of a real-world school setting and the different types of context-relevant outcomes (e.g., achievement test performance and in-class behavior) that might be effected by working memory training.

Arguably, the ideal way to establish construct validity among multiple measures is to assess transfer effects via latent factors within a structural equation modeling framework [21]. However, the measures we used varied across a range of dimensions and therefore did not lend itself easily to latent modeling. Instead, we examined the interrelations between the different measures in order to provide indirect information about the construct validity of the transfer tasks. Ideally, we should see that the different measures are correlated and that intervention effects are obtained across a wide range of correlated tasks.

A related issue that has thus far has received little attention concerns the relation between training and transfer tasks. Improvement in a training task can be expected for those transfer tasks that tap the same underlying ability as the training task [22]. Ideally, the magnitude of any such improvement is a monotonic function of the strength of the relation between the training and transfer tasks. To be concrete, knowing the strength of the relation between performance in the training task (e.g., assessed at the beginning of training) and performance on a given transfer task should allow us to predict the extent to which training-related gains in working memory will result in gains in the target ability, at least as an upper-bound estimate. The empirical relationships between training and transfer tasks may be clouded by other factors, such as strategy effects. Nevertheless, the presence or absence of meaningful relationships between training and transfer tasks is useful for evaluating the overall pattern of training gains [22], [23]. In previous work, such correlational information would have been difficult to interpret, because of small sample sizes (usually around 20 subjects per group). Therefore, in the current study we assessed training and transfer in a relatively large sample of more than 100 children in both the experimental and control groups.

### Current Study

To address the issues detailed above, we sampled a large group of children from several schools and randomly assigned classrooms of them to working memory training and control groups. Assessments included pretest and posttest performance in both working memory and standard academic achievement measures, as well as teacher ratings of problematic behavior in the classroom. The experimental group completed an intensive computerized training program, modeled after a previously successful procedure [19]. The training was delivered in the school context during normal class time, while students in the control condition experienced regular instruction. Thus, with this design we can address the question to what degree working-memory training produces greater benefits than spending about the same amount of time with normal classroom activities.

## Methods

### Ethical Standards

This project was reviewed by the Institutional Review Board of the Oregon Center for Applied Sciences. Since this study compared instructional techniques in an educational setting, the IRB determined it to be exempt under CFR 46.101(b)(1).

### Subjects

282 children from 11 third grade classrooms across five different schools within the same school district completed the study. Sixty percent of students in the school district receive free or reduced lunch.

Children were randomly assigned unique ID codes for use on pre- and post-test measures and for program use tracking. Each school contributed between one and three classrooms. Within each school, entire classrooms were assigned to either the intervention (six classrooms, *n* = 156) or control condition (five classrooms, *n* = 126), such that each school contributed at least one classroom to either condition, except for one school with only one participating classroom.

### Assessments

Baseline reading comprehension and mathematical reasoning were assessed one week prior to intervention start using the Wechsler Individual Achievement Test – II [24]. The children completed the tests using pencil-and-paper in groups of 15 or –fewer, with testing supervised by two researchers. The Reading Comprehension subtest (WIAT-Read) was started on item 20 (based on difficulty level appropriate for 3^rd^ graders) and students were given 15 minutes to complete as many questions as possible (max score = 45). Following a three minute break, children were then given 10 minutes to solve Mathematical Reasoning subtest items (WIAT-Math), starting with item 21 (max score = 30). These tests were re-administered in the week following WM training to assess potential transfer effects.

We obtained state standardized test scores administered within 2 weeks prior to the start of the intervention period for each student. These consisted of the Easy Curriculum-Based Measurement [25], [26] tests of 3^rd^ grade reading fluency (CBM-Read) and math assessments (CBM-Mat). The EasyCBM tests are used in Oregon to monitor progress on the level of schools and to identify children with potential learning problems. The reading fluency test scores the number of words students were able to read correctly from a story within 1 minute. The math component consists of a computerized assessment of grade-adequate math multiple-choice problems such as locating fractions on a number line or completing simple number sequences. Follow-up scores for the same tests were obtained approximately 8 weeks after completion of the WM training.

In addition to these paper-and-pencil assessments, six children were randomly selected from each classroom for individual testing (intervention: *n* = 36, control: *n* = 30) with the Automated Working Memory Assessment [27]. Four of the AWMA tests were administered: Listening Recall for verbal short-term memory, Counting Recall for verbal working memory, a measure of the central executive, and “Odd One Out” and “Mister X” for visuo-spatial working memory. These tests were administered in the week prior to WM training and again for the same children in the week following WM training.

We also asked teachers to rate each student's behavior using the Working Memory Rating Scale [28]. This is a 20-item scale that asks teachers to rate how typical certain behaviors are for each student. Examples include “The child raised his hand, but when called upon, had forgotten his response”, or “She lost her place in a task with multiple steps”.

### Intervention Procedure

Children in the intervention group spent 20–30 minutes each day (Monday through Friday, excluding occasional school closure days) for four weeks using an interactive working memory (WM) training program. We dropped children who participated in fewer than 8 sessions (7 children). The average number of sessions for the remaining children was 17.13 (SD = 2.55). The program was administered in a computer lab at the school site. Children were monitored to keep them on task but the program itself was self-paced. Children wore headphones to hear their own program directions and feedback without distraction from neighboring children. Each student logged into the program with his or her own unique ID code. All testing parameters were automatically logged by the program.

The program began with an introduction presented by two animated characters. They walked the student through one cycle of the WM task, and then gave feedback as the student tried two more cycles. After the introductory training, the characters provided no more prompts, but gave positive feedback on correct trial sets. Each student had a virtual locker and was rewarded with stickers for their locker for every correct WM training trial. Every 5 minutes the student was given a break to view their locker and the stickers they had earned. For increased engagement, for every 20 correct trials, the student unlocked a new “wallpaper” for their locker with a new set of stickers to go with it.

**Figure 1:**
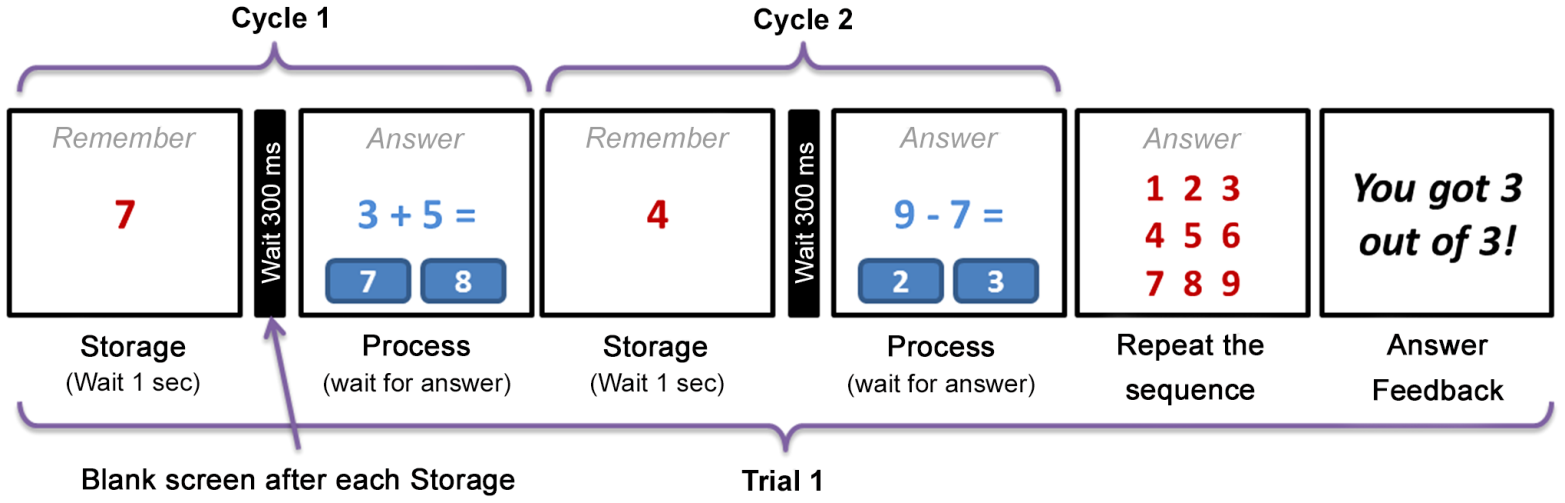
Example of a training task item with two memory/math cycles.

The training task was modeled after that used by [19]. Task delivery occurred in a game-like format, and as shown in Figure 1 involved a series of numbers to memorize (storage) interspersed with math tasks (process) at increasing levels of complexity. One cycle consisted of one storage number presented for one second, a 300 ms blank screen, and a math task with a choice of two possible answers which remained on screen until the student responded. One trial consisted of a varying number of cycles followed by a number array on which the student selected the storage numbers in the original sequence. The program started with a trial of two cycles. For every two correct trials (storage number recall and 75% of math answers correct) the program added one cycle to the trial series. Every time the student missed either the recall sequence or had more than 25% errors on the math problems, the program reduced the trial sequence by one cycle, with a minimum of two.

The math tasks started with the addition of two single digits problems. If the student had >75% accuracy on the math tasks over a 5 minute session, the task increased in complexity to missing addend problems (*e.g.* 9+_ = 16), then to addition of three single-digit numbers. The complexity decreased with 3 sequential incorrect problems.

During the time when the children in the experimental group were administered the intervention task, children in the control group received regular instruction in their classrooms. Thus, with this design we can test to what degree the intervention leads to training/transfer gains beyond instruction as usual for the same amount of time as spent on the intervention. Students in the control group were not explicitly notified about their status. However, as there were experimental and control groups in the same schools (except for one) we cannot rule out that children's or teacher's knowledge of their status within the design may have affected outcomes, a point we will return to in the General Discussion.

## Results

We first document practice effects in the working memory intervention task, and then report the relations between pretest, training, and transfer performance to verify the construct validity of both the training task and our transfer measures. Third, we will report the transfer effects and finally examine the relation between practice task performance/gains and transfer gains.

### Training

Children practiced the intervention task within the school setting. Determining the amount of within-task experience each session is difficult because—due to the adaptive regulation of the number math/memory elements per trial—the number of math problems that were presented varied with the level of success with the task. For children who completed more math/memory cycles, each encoding/retrieval trial took longer because they encountered more difficult math problems. As a result, each cycle took more time and thus fewer of them could be completed during the practice session. Therefore, we used the cumulative number of math/memory cycles encountered per session (M = 76.93, SD = 27.79) as a proxy of the actual on-task experience. For example, for a student whose average number of adaptive math/memory cycles in a particular session is 2 (i.e., they solved two math problems before given the number recall task), this would amount to about 38 recall problems for this session.

**Figure 2:**
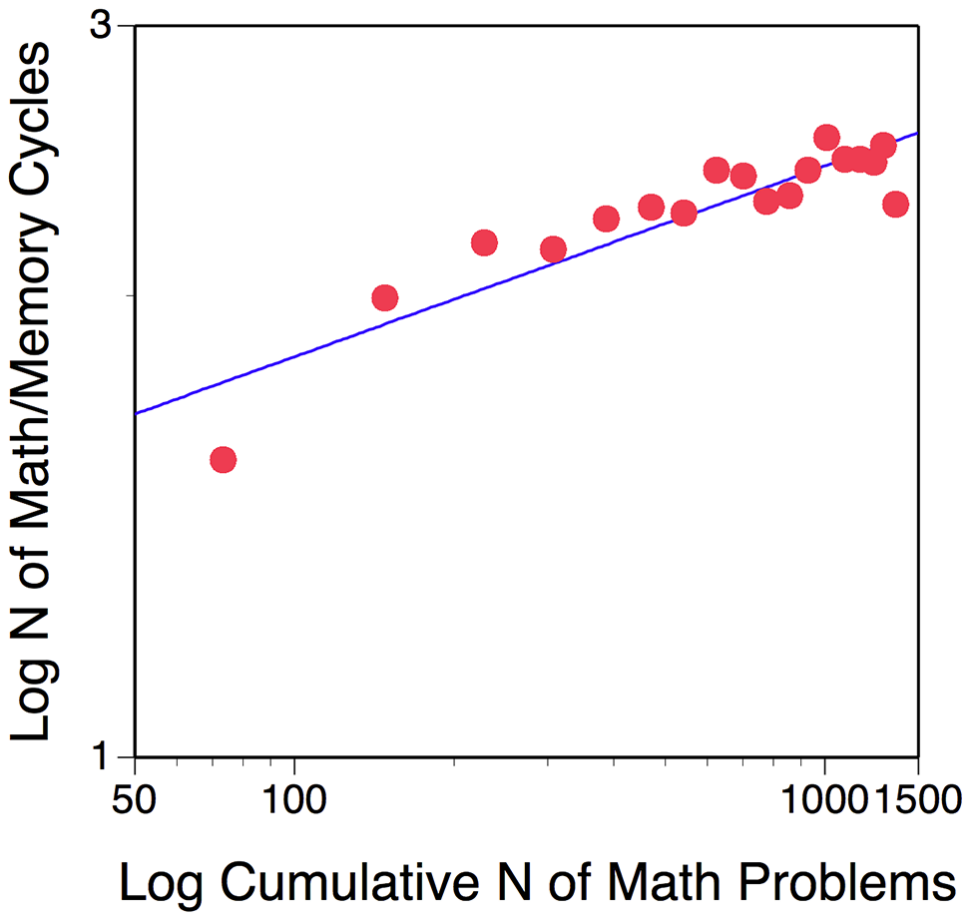
Log of math/memory cycles per session as a function of log of cumulative math problems solved in the context of the working-memory training task. The line represents the best-fitting linear function, which in log/log space is equivalent to the power function with untransformed scores.

Figure 2 shows the number of math/memory cycles for each of 18 practice sessions (which 75% of all children had completed) as a function of actual experience with the task (i.e., *N* of arithmetic problems processed). For example, in Session 1 subjects performed about 74 math problems and achieved an average number of 1.56 math/memory cycles. By the time subjects had reached Session 15, they had performed on average of 1167 math problems and achieved 2.4 math/memory cycles.

Practice trajectories often conform to a power law, which forms a linear function when represented in log-log space (i.e., log of *N* of adaptive cycles and *N* of problems). As apparent from Figure 2, the average training data are fit by the Power function relatively well (R^2^ = 82%). The underestimation of Session-1 results is a relatively common result in the training literature and can be attributed to factors such as initial familiarization with the task and—in this particular context—also to the fact that the adaptive process starts each child off with a cycle length of 1.

Using the *lmer* function of the statistical package [29] we fitted log N of cycle scores for each session as a function of the log of cumulative N of math problems across all sessions. The intercept and the slope of the linear function were included as both fixed effects and random effects. The learning rate was .101 (*SE* = .010), *t* = 8.04. The intercept (.016, *SE* = .021) did not differ from zero, *t* = .5. Thus, children clearly benefitted in terms of their ability to remember number sequences interspersed with solving math problems.

A learning rate of about .1 translates into a mean increase of .7 math/memory cycles as a function of the on average 1182 math problems presented across all practice sessions. Below, we will use this information when we examine the relation between children' training gains and transfer gains.

### Construct Validity of Transfer Measures and Relation with Training Task

With our transfer measures we wanted to capture the targeted ability (i.e., working memory), but also potential transfer to school-relevant reading and mathematics achievement and teacher ratings of behavior. Because, as noted earlier, our transfer measures were dictated in part by practical constraints of conducting this study in a school context, it was important to verify that there are meaningful relations between the different tasks.

Table 1 shows all relevant correlations for the pretest scores of the two paper and pencil working memory (WIAT Math and Reading Tests), the two standard math and reading achievement measures (CBM Math and Read), the behavioral teacher rating, and the computerized working memory measure (AWMT). Because of variations in attendance across test occasions, the sample size among the first five measures varied from 244 to 291; the sample size for the computerized measure varied between 56 and 63 (32 for the correlation with the training task); the sample sizes for the training task were between 137 and 150). As apparent, there were moderately large relations among all transfer measures (all *p*s<.01). There was also some meaningful, content-specific variation, with the within-domain WM/achievement correlations (e.g., math with math) being somewhat higher than the across-domain correlations (e.g., math with reading).

**Table 1:** Intercorrelations among all pretest measures and Session-1 performance in the training task for the experimental group.

	WIAT Read	CBM Math	CBM Read	Teacher Rating	AWMT	Session 1 Training
WIAT Math	.584	.619	.557	−.502	.535	.510
WIAT Read		.421	.686	−.482	.423	.242
CBM Math			.405	−.483	.508	.510
CBM Read				−.468	.373	.382
Teacher Rating					−.365	−.349
AWMT						.523

Notes. For the Teacher Rating measure low scores indicate better performance.

More important, the first-session performance in the training task showed highly systematic relations with all working-memory tasks, the achievement tasks, and the teacher rating scale. These relations were stronger for the math measures than for the reading measures, likely reflecting the fact that the training program used simple math problems for the processing component.

We can go one step further and regress pretest performance for each of the transfer measures on session-1 performance in the training task. The resulting coefficients can be used to provide an upper-bound estimate of the degree to which a given improvement in the training task should translate into benefits in the transfer task. For example, the unstandardized coefficient relating the training task session-1 performance to the WIAT Math task is 3.5. Thus, an across-sessions increase in the training task by .7 adapted cycles (the average gain across practice) should lead to an increase of 2.5 (i.e., .7×3.5) more problems solved correctly on the WIAT Math task, if the training task gains actually reflect improvements in working memory. We then use the pretest standard deviation to compute an estimated effect size, which in this case is d = .62. We can compare this value with the empirical effect size, as described in the next section.

### Transfer

Next we turn to the question to what degree training produced transfer effects on academically relevant abilities. Because children were recruited from five different schools, we used mixed linear modeling to estimate school-related variance. Specifically, we estimated the pretest-posttest difference as a within-subject fixed effect, the experimental condition as a subject-level fixed effect, and as the theoretically critical effect we included the interaction between these two predictors. We included the pretest-posttest contrast also as a random effect and we allowed intercepts and coefficients for random effects to vary both across schools and across children within schools. This procedure also allowed us to use all available data, even when information from one of the two measurement occasions for a particular measure or student was missing. For sake of completeness, we also report in Table 2 the raw pretest/posttest scores for all transfer tasks separately for the experimental and the control group.

**Table 2:** Raw mean scores (SD) for all pretest and posttest measures, separately for the training and the experimental group.

	Pretest		Posttest	
	Mean	SD	Mean	SD
*Experimental Group*				
WIAT Math	9.95	4.10	10.93	4.11
WIAT Reading	18.09	8.53	24.39	9.91
CBM Math	33.76	6.46	38.65	5.35
CBM Reading	118.85	43.30	130.94	41.68
Teacher Rating	18.20	16.88	13.51	15.35
AWMT	24.23	7.13	31.59	11.24
*Control Group*				
WIAT Math	10.05	3.82	10.85	4.09
WIAT Reading	19.08	8.48	24.39	9.26
CBM Math	33.50	6.23	37.53	5.67
CBM Reading	118.70	41.70	133.10	40.76
Teacher Rating	10.60	13.73	10.821	13.59
AWMT	21.26	6.23	25.94	7.28

Note. The model-based effects shown in Figure 3 integrate information from all subjects (even in case of missing values) as well as the grouping by schools and therefore differ slightly from the raw-score results presented here.

**Figure 3:**
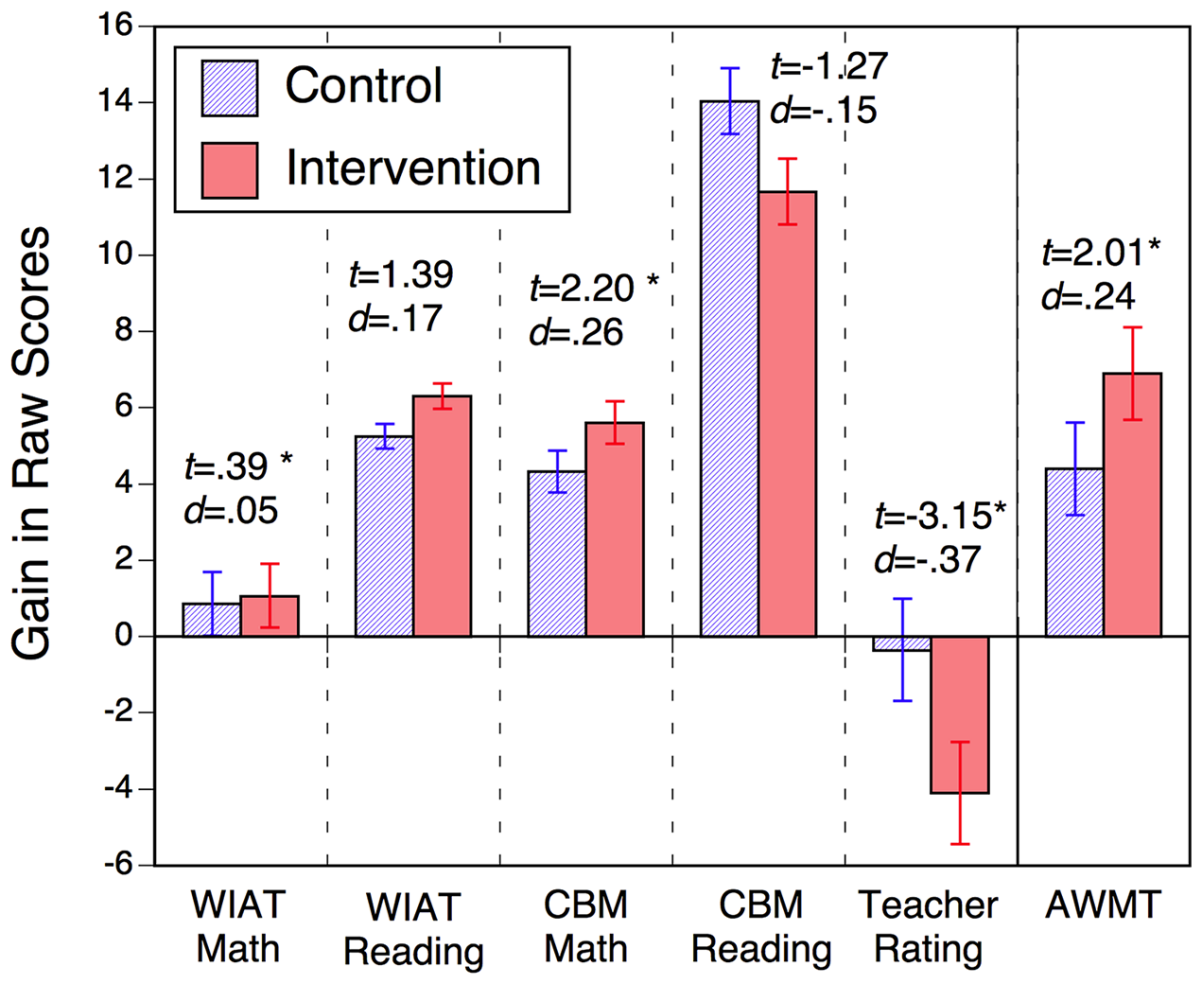
Gains in raw scores for all transfer measures for the control and the intervention group, estimated through the multi-level model, as well as *t*-values and corresponding effect sizes (*d*) of the between-group differences.

Figure 3 shows raw score gains for the control and the intervention conditions for all measures, as estimated through the mixed linear models. Recall that the computerized AWMA test was only assessed for a subgroup of children, whereas the remaining measures were assessed in the complete sample. Also, note that the Teacher Rating is of problem behaviors, and therefore a reduction in score reflects improved classroom behavior. Across all measures, except for the Teacher Rating, there were significant gains for both the control and the intervention conditions. Moreover, these gains were numerically larger for the intervention than the control condition for each measure, except for the CBM Reading score. However, only the AWMA, the CBM Math, and the Teacher Rating showed significant condition differences.

**Figure 4:**
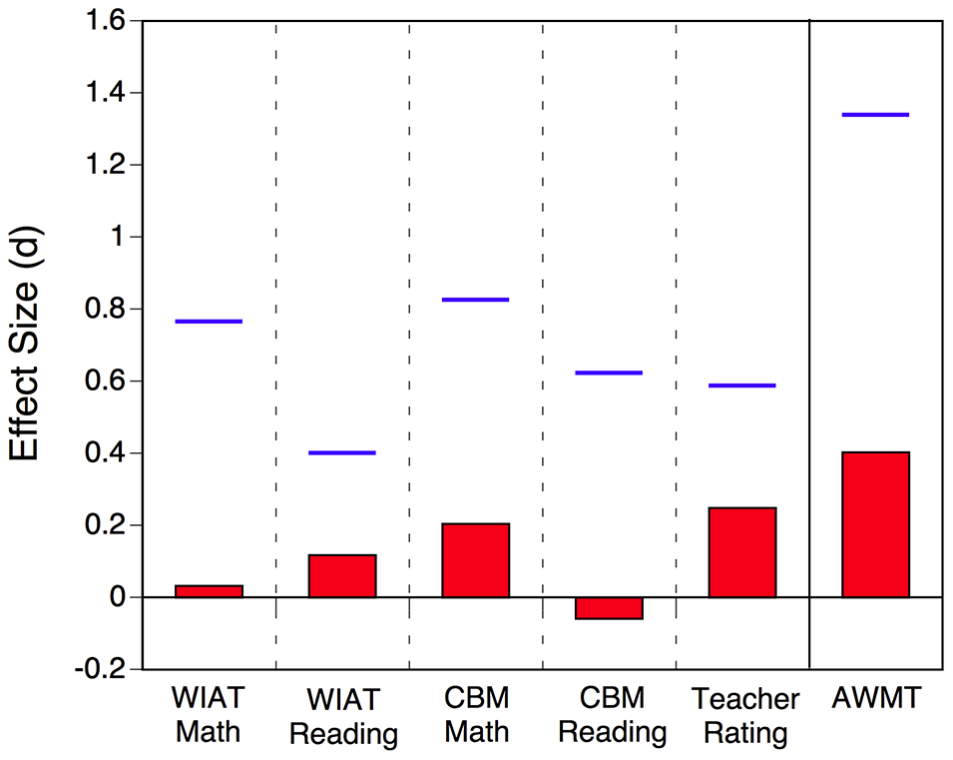
Bars represent empirical effect sizes for each transfer measure and horizontal lines represent the estimate of the expected effect size based on the relation between session-1 training-task performance and pretest transfer-task performance. Both empirical and estimated effect sizes are shown in reference to the pretest standard deviation of the training group for each measure. The purpose of these effect sizes is to allow a comparison between expected and actual effects; standard effect sizes are shown in Figure 3. In each case, a positive effect size reflects a transfer benefit.

Figure 4 shows the intervention effects in terms of effect sizes referenced to the pretest standard deviation of the training group, along with an estimate (horizontal line) of the expected effect size based on average improvement in the training task and the strength of the relation between training task performance (assessed during the first session) and each transfer measure at pretest (see previous section). As apparent, the observed effect sizes are substantially smaller than the predicted effect sizes. Additionally, the expected pattern of largest gains on the AWMA, followed by the two math tests, and then the two reading tests was not found; expressed as a correlation the similarity between expected and observed gain profiles was actually negative *r* = .02.

Thus, while there is some evidence for modest transfer effects, these effects differed from the effects that would be expected based on the relations between training and transfer tasks as established through the individual differences analyses (see Table 1).

### Training-Transfer Relationships

**Figure 5:**
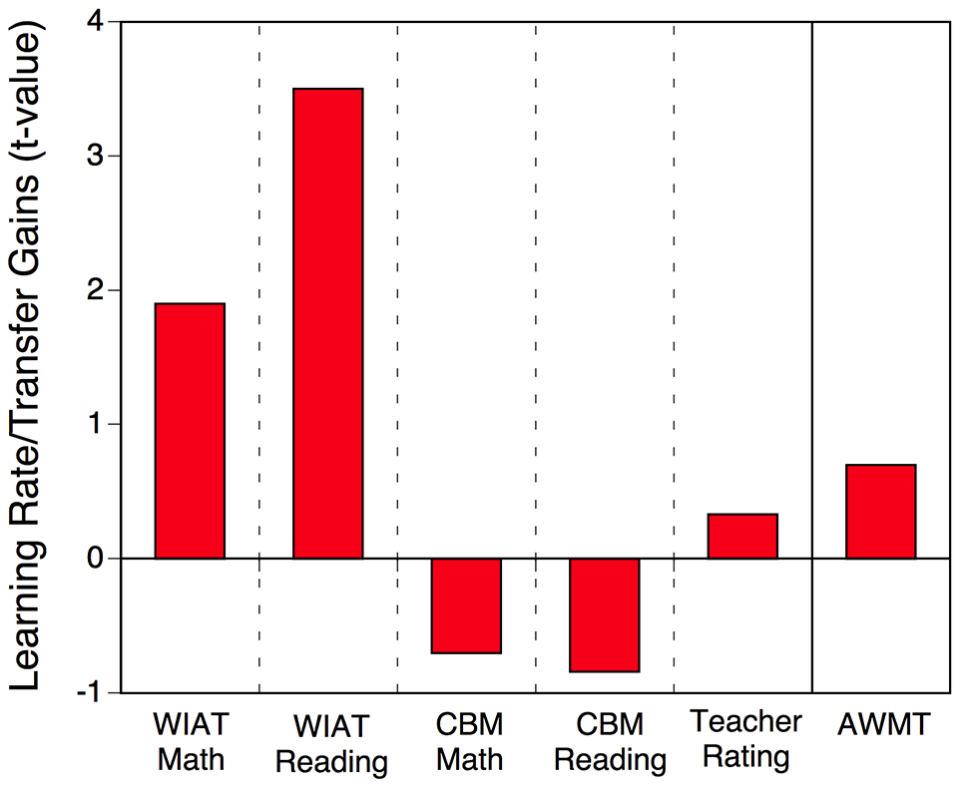
*t*-values representing the strength of the relation between individual students' total gains in the training task and pretest-posttest gains in each of the transfer tasks. In each case, a positive *t*-values indicates that a larger learning rate is associated with greater transfer benefits.

Within the intervention group we can also examine the degree to which training gains predicted transfer gains. Training gains were computed for each individual student in the experimental group using his/her estimated learning rate and intercept and the actual amount of training (i.e., number of training cycles they completed across sessions). We used a hierarchical linear model to predict each student's pretest and posttest scores for each of the transfer measures using the pretest/posttest, individual training-task gains, and the interaction between these two variables as fixed-effect predictors. This interaction captures the degree to which pretest-posttest transfer gains are related to the gains in the training task. If training gains were the causal factor behind transfer gains, the two should be related, in particular for measures with significant intervention effects. Figure 5 shows that a trend emerged for WIAT Math and a significant relation was found for WIAT Reading. However, neither of these had shown reliable intervention effects comparing the experimental and control groups. At the same time, there were no significant training-transfer effects for any of the measures that did show reliable intervention effects.

This analysis also provides information about the degree to which gains in the training task and overall level of performance in the transfer measures (i.e., averaged across pretest and posttest scores) are correlated. Interestingly, this relation was positive and either close to significant or significant for almost all measures, AWMA: *t* = 1.86, WM Math: *t* = 4.0, WM Reading: *t* = 3.15, CBM Math: *t* = 3.15, CBM Reading: *t* = 2.14, Teacher Rating: *t* = 3.24. This result indicates that, while the relation between training and transfer gains is tenuous at best, there is a relation between overall working memory/academic performance (i.e., averaged across pretest and posttest) and the amount of gain in the training task. In other words, higher achieving children gained more on the training task than did lower achieving children.

## Discussion

Our main goal was to determine to what degree working memory training within a regular school setting produces beneficial effects that extend to school-related achievement and in-class behavior over and above the benefits of “standard classroom treatment”. Important aspects of our study were (a) a large sample of close to 300 children assessed within a classroom setting, (b) the use of a relatively wide range of both standard working memory and math and reading achievement tests, and (c) our use of the empirical relations among training and transfer tasks to produce reasonable predictions about expected transfer gains as a yardstick for empirical transfer effects.

We did find substantial improvements in training task performance across the approximately 17 sessions. We also found sizeable relations between the initial training task performance and performance across the different transfer tasks. Thus, assuming that the individual differences relations between training and transfer tasks represent the overlap in functions between these tasks, we should obtain transfer gains.

Yet, evidence regarding transfer gains was mixed. We found a significant transfer effect for the computerized working memory test battery, but no comparable effects in any of the other tasks in this subgroup of children. In the complete sample, we obtained significant, small effect-size transfer gains for the CBM math-achievement test and in the teacher ratings (see Figure 3), but no effects in the two working memory and the reading-achievement tests. Also, in each case actual transfer gains were by an order of magnitude smaller than the gains predicted on the basis of the empirical relation between training and transfer tasks. Moreover, there was little resemblance between the profile of the effect sizes predicted from the observed relation between training and transfer tasks and the actual transfer effects.

Similarly, within the intervention group, the profile of individual differences relations between training gains and transfer gains was not consistent with the profile of transfer gains. The only significant relation was obtained for the working-memory reading task, which showed little evidence for a transfer gain. In theory, if training related improvements in working memory were causal factor underlying any transfer gains, one would expect that transfer effects to increase as a function of the size of the training-gain/transfer-gain relation [22]. We did not find evidence for this predicted pattern. In total then, these results do not provide strong evidence in favor of substantial and educationally meaningful transfer gains that go beyond the benefits achieved through regular classroom instruction.

There may be task-specific or strategy-specific factors that affect the different types of relationships between training and transfer performance or learning we presented here. Thus, the rather unimpressive pattern of relationships by itself may not be a strong reason to dismiss the hypothesis that working memory training produces broad benefits. This hypothesis, however certainly is not helped by the fact that neither the pattern of mean transfer effects nor the pattern of training-task/transfer task-relationships is consistent with the hypothesis of meaningful, generalized benefits.

It is also important to note that compared to most other training studies in the literature our sample sizes relatively are large. Therefore, the lack of coherent and significant transfer effects cannot be attributed to low statistical power. By the same token, with our large sample size, even relatively small effect sizes with little educational significance could reach statistical significance.

Interestingly, we did find that in almost all cases the overall ability (averaged across pretest and posttest) was a reliable and positive predictor of gains in the working memory training task. In other words, at least in the context of this particular task it seems that high-ability children profited more from training than low-ability children. Thus, even if working memory training did produce general benefits, if anything, it would increase rather than compensate for existing differences.

### Limitations

We used only one of several possible training procedures and therefore we cannot make strong claims beyond this particular program. However, we selected this training task because it has produced generalized effects in past research [19] and because it could be easily used even with younger children. Also, in terms of its adaptive testing regimen, training intensity, and demands in terms of both storage and processing, our training program shares characteristics with many other training paradigms that have been used in the literature.

Ideally, different abilities are assessed in terms of multiple indicators in order to establish latent constructs through structural-equation modeling. In principle, training effects that occur on the level of the latent construct cannot be easily explained in terms of superficial strategy effects [20]. Given the constraints of the school setting, it was not possible to conduct the necessary, intensive pretest-posttest assessment. However, we documented moderate-to-substantial relations between the different transfer tasks, as well as between the training task and transfer tasks. In light of these relations, the small transfer effect sizes and their inconsistency across tasks is particularly striking.

Finally, an important limitation of the current work is that because testing and training occurred within a regular school setting it was not feasible to use a double-blind design with an active (placebo) control group. This leaves the possibility that observed transfer gains result from expectancy/placebo effects [20]. Obviously, this would be of greater concern had we actually observed pedagogically significant training benefits. In addition, one could argue that information about the relationships between training gains and transfer effects provides a plausibility check of potential, “true” transfer gains. If the intervention leads to improvements in working memory capacity then gains on the transfer tasks should vary based on the extent to which these tasks require the same working memory skills as the training task. In contrast, a potential placebo effect should influence performance all transfer tasks in an indiscriminant manner. In this context it is noteworthy that the teacher ratings of problem behaviors showed the largest empirical transfer effects, even though only moderate transfer effects were predicted (based on relationships with the training task), and the individual differences correlations with learning rates were (slightly) in the opposite direction (i.e., greater learning rates leading to more problem behavior). Adding the fact that the ratings were made by teachers who could not be blinded with regard to students' status within the study design, this pattern suggests that the relatively large transfer gains for this measure probably resulted from expectancy effects [30].

## Conclusions

Based on recent work, there was reason to believe that intensive and adaptive working memory training is a viable vehicle to boost children' working memory functioning, and as a result, achievement in working-memory based, school-related skills. However, despite the fact that we (a) used a relatively standard working memory training task, (b) tested a large sample in a variety of transfer tasks, and (c) could document the construct validity of both the training and transfer measures, we found very little evidence that would confirm the hypothesis that working memory training has generalized effects on intellectual functioning and/or academic performance. It is possible that different training tasks or more intensive training may eventually show pedagogically relevant gains. However, the current results join a growing chorus of studies that all report disappointing generalized effects from intense working memory training [31], [32]. In the absence of positive evidence from methodologically sound studies [21], attempts to use working memory training programs in a pedagogical/clinical context should be considered with great caution.
